# Supplementary material for: A whole-animal phenotypic drug screen identifies suppressors of atherogenic lipoproteins
Source: eLife. 2026 Aug 27;14:RP105314. doi: 10.7554/eLife.105314 (PMC13521566; doi:10.7554/eLife.105314)
Supplement: Supplementary file 2 [file elife-105314-code1.zip › JHDL_primary_result/Unknown/Unknown_JHDL_17_015_055_2019_09_13_12_43.pdf]

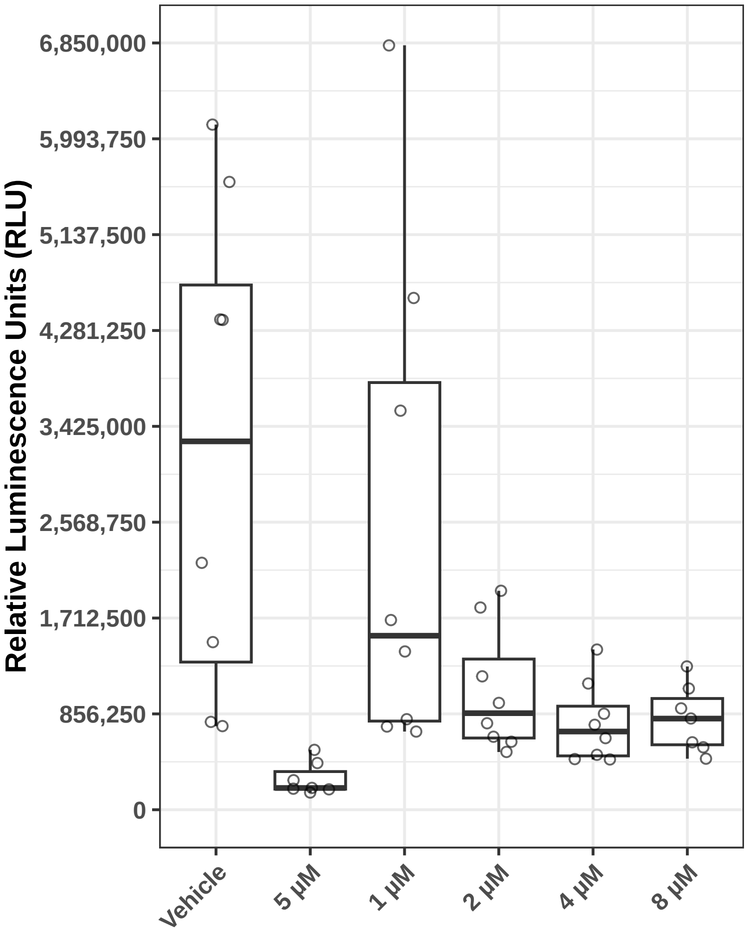
Relative Luminescence Units (RLU)
6,850,000
5,993,750
5,137,500
4,281,250
3,425,000
2,568,750
1,712,500
856,250
0
Vehicle
5 μM
1 μM
2 μM
4 μM
8 μM
